# *DeepCount*: In-Field Automatic Quantification of Wheat Spikes Using Simple Linear Iterative Clustering and Deep Convolutional Neural Networks

**DOI:** 10.3389/fpls.2019.01176

**Published:** 2019-09-26

**Authors:** Pouria Sadeghi-Tehran, Nicolas Virlet, Eva M. Ampe, Piet Reyns, Malcolm J. Hawkesford

**Affiliations:** ^1^Plant Sciences Department, Rothamsted Research, Harpenden, United Kingdom; ^2^Phenotyping, Near Infrared and Research Automation Group, Limagrain Europe, Chappes, Netherlands

**Keywords:** wheat ear counting, crop yield, deep learning in agriculture, semantic segmentation, superpixels, phenotyping, automated phenotyping system

## Abstract

Crop yield is an essential measure for breeders, researchers, and farmers and is composed of and may be calculated by the number of ears per square meter, grains per ear, and thousand grain weight. Manual wheat ear counting, required in breeding programs to evaluate crop yield potential, is labor-intensive and expensive; thus, the development of a real-time wheat head counting system would be a significant advancement. In this paper, we propose a computationally efficient system called *DeepCount* to automatically identify and count the number of wheat spikes in digital images taken under natural field conditions. The proposed method tackles wheat spike quantification by segmenting an image into superpixels using simple linear iterative clustering (SLIC), deriving canopy relevant features, and then constructing a rational feature model fed into the deep convolutional neural network (CNN) classification for semantic segmentation of wheat spikes. As the method is based on a deep learning model, it replaces hand-engineered features required for traditional machine learning methods with more efficient algorithms. The method is tested on digital images taken directly in the field at different stages of ear emergence/maturity (using visually different wheat varieties), with different canopy complexities (achieved through varying nitrogen inputs) and different heights above the canopy under varying environmental conditions. In addition, the proposed technique is compared with a wheat ear counting method based on a previously developed edge detection technique and morphological analysis. The proposed approach is validated with image-based ear counting and ground-based measurements. The results demonstrate that the *DeepCount* technique has a high level of robustness regardless of variables, such as growth stage and weather conditions, hence demonstrating the feasibility of the approach in real scenarios. The system is a leap toward a portable and smartphone-assisted wheat ear counting systems, results in reducing the labor involved, and is suitable for high-throughput analysis. It may also be adapted to work on Red; Green; Blue (RGB) images acquired from unmanned aerial vehicle (UAVs).

## Introduction

Yield is composed of three components: number of ears per unit area, number of grains per ear, and grain weight, some which may be estimated during the growing season. The early estimation of preharvest yield allows breeders more rapid germplasm assessment and enables farmers to adjust cultivation practices to optimize production. Manual counting protocols have been the only way of calculating the number of ears per square meter (ears/m^2^). Breeders can identify and count wheat spikes visually; however manual counting of wheat spikes is labor-intensive and time-consuming. In addition, these tasks may need to be performed on many thousands of cultivars, which is likely to introduce human error into the obtained data. An ideal alternative would be the development of automated systems operating under field conditions. Recent advances in automated data acquisition systems (Busemeyer et al., 2013; Virlet et al., 2016; Kirchgessner et al., 2017) allow a high spatial sampling due to the rapidity of the image acquisition process, which enables all possible measurements of crop growing status. Even though the ability to acquire data is relatively fast and easy, challenges remain in terms of the data mining of images. Computer vision offers an effective choice for analyzing high-throughput image-based phenotyping due to low-cost (relative to man-hours invested into manual observations) and the requirement for minimal human intervention. Although current computer vision systems are increasingly powerful and capable, they still need to overcome the difficulties associated with images acquired under field conditions. Environmental noise causes major challenges for computer vision-based techniques in identifying objects of interest, such as wheat spikes. Some challenges include the following: (*i*) plant movements and/or stability of handheld cameras may cause blurred images; (*ii*) dark shadows or sharp brightness may appear in images due to natural condition and light variations in the field even though a camera is set to auto exposure; (*iii*) overlaps between ears due to a floppy attitude of the ears may also cause additional difficulties, especially with the presence of awns in some cultivars; and (*iv*) spikes in different varieties change significantly through development stages, as spikes show only little similarity between the early and later growth stages.

Several studies have utilized image-based automatic wheat ear counting for early evaluation of yields (Cointault and Gouton, 2007; Fernandez-Gallego et al., 2018; Cointault et al., 2008b). These methods have relied on image data extraction techniques related to characteristics of color, texture, and morphological operations. Cointault et al. (2008b) proposed a mobile platform to acquire data where visible images were taken by a digital camera located vertically above the field of view using a tripod. The field of view is a closed system delimited by a black matte frame to control variabilities in illumination and weather conditions. The proposed framework creates a homogeneous environment and blocks unwanted image effects. Subsequently, the authors improved their platform by collecting images in different lighting conditions without any structure blocks (Cointault et al., 2008b). The main drawback is the restricted data acquisition pipeline required for the system to operate. For instance, prior knowledge of the environment is required to achieve an optimum result; moreover, even with the current restrictions, only a small number of images were selected based on which the authors felt presented “good illumination.” In a similar approach (Cointault and Gouton, 2007; Cointault et al., 2008a; Fernandez-Gallego et al., 2018), a supervised classification method was proposed to distinguish three classes of leaves, soil, and ears. In the end, morphological operations were applied for counting the number of blobs (potentially ears) from the binary image with the preassumptions of the shapes of the ears. Each pixel is represented by color and texture properties. As suggested, a hybrid space is constructed to address a sensitivity of color properties to the intensity variations in an image. The method has been tested on a limited number of wheat varieties without awns with a low level of wheat ear density; nonetheless, no evaluation was carried out to validate the accuracy of the proposed method with the manual measurements. In another study, Fernandez-Gallego et al. (2018) applied Fourier filtering and two-dimensional discrete Fast Fourier transform (FFT) (Cooley and Tukey, 1965) to distinguish wheat ears from the background. The approach performs, in three main steps of high-pass filtering, thresholding, and mathematical morphology, operations to eliminate “nonwheat” pixel groups, which are small and scattered. The threshold is predefined by a user to determine if pixels should be identified as foreground (ears) or background (leaf, soil, etc.). The drawback is that a wrong choice of the threshold value may result in distortion and low performance of the whole system in different environments. Finally, Zhou et al. (2018) proposed a twin-support-vector machine segmentation method to segment wheat ears from visible images. The method relies on the hand-engineered features, including color, texture, and edge histogram descriptor. The images were collected from the side at 45 degrees above the horizontal because color and texture were suggested being typically more substantial from this perspective.

At the core, the success of any of the current state-of-the-art methods crucially depends on the feature representation of the images. While the aforementioned methods use handcrafted features to represent images by encoding of various features including corners, edges, texture, and color schemes, the features are tailored to a specific condition, and their effectiveness is inherently limited as these approaches mainly operate at the primitive level. Unlike conventional feature extraction techniques, which often use shallow architecture and solely rely on human-crafted features, relatively new learning-based methods based on convolutional neural networks (CNNs) show promising results for visual analysis. CNN models attempt to model high-level abstractions in images by employing deep architectures composed of multiple nonlinear transformations (Lomonaco, 2015; Schmidhuber, 2015). In CNN, features are extracted at multiple levels and allow the system to learn complex functions that directly map raw sensory input data to the output, without relying on hand-engineered features using domain knowledge. The convolution is an operation of applying the filter on a single color image to enhance some of its features. One-to-one convolutions take a single image as an input and return a single image as an output. However, in CNN, different kinds of convolutions exist. For instance, in one-to-many convolutions, a single input image is passed to *k* filters; then each filter is used to generate a new output image. Alternatively, in many-to-many convolutions, there are *n* inputs and *m* outputs where each output image is connected to one or more input images characterized by *k* filters (Lomonaco, 2015). Potentially, this capability makes the deep neural network more robust to different types of variations in digital images. As a result, the model can adapt to such differences and has the capacity to learn complex models.

In recent years, CNNs have shown usefulness in a large variety of natural language processing and computer vision applications, including segmentation and image classification, and often surpassed state-of-the-art techniques (Krizhevsky et al., 2012; Mikolov et al., 2013; Lomonaco, 2015). Despite the promising outcomes of deep learning in computer vision, there are some limitations in implementing a deep neural network. Deep learning approaches are usually computationally intensive, and their performance relies on the quantity and quality of training datasets. In most cases, for deep learning to show great advantages, training datasets of tens of thousands to millions are required (Deng et al., 2009; Ubbens et al., 2018). Having a large training dataset provides deep learning models with extensive variety, which leads to an effective learned representation as a result. Deep neural networks (DNN) are an area of active research, and applications to plant research are still in the early stages. There are few deep learning applications successfully applied in the field of image-based plant phenotyping (Pound et al., 2017; Madec et al., 2019). The small body of existing applications includes plant disease detection on leaf images (Mohanty et al., 2016), rice panicle segmentation (Xiong et al., 2017), leaf counting in rosette plants (Ubbens et al., 2018), wheat ear counting (Madec et al., 2019), and localizing root and shoot tips (Pound et al., 2017).

This study utilizes a novel visual-based approach based on linear iterative clustering and deep CNNs to identify and count the number of wheat spikes. The proposed method can also calculate the number of wheat ears per square meter when a ground standard is present within the image. The proposed method, called *DeepCount*, alleviates the limitations and lack of separability inherent in existing wheat ear-counting methods and minimize the constraints of capturing digital images taken under natural outdoor environments. The approach presented will pave the way for computationally efficient and significantly faster approaches compared to the manual techniques, leading to reducing the labor involved and enabling high-throughput analysis.

## Materials and Methodology

In this study, we explore the feasibility of automatically identifying wheat spikes under natural in-field conditions based on a completely data-driven framework. The main contributions of the work can be summarized as follows:

- Building a high-quality dataset of annotated spikes and utilizing them to train our CNN model.
- Developing a deep learning model called *DeepCount* that can learn from the training dataset and then identify and segment spikes from different wheat cultivars (awns and no awns).
- Demonstrating that the constructed model can automatically quantify the number of spikes within visible images under natural field environments and calculate the number of ears per square meter when a ground standard is present.

**Figure 1:**
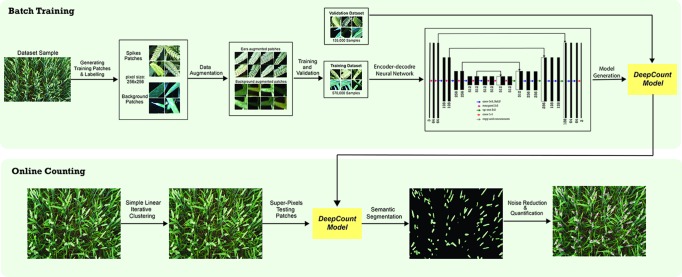
Schematic representation of the *DeepCount* method.

Quantification of spikes may be achieved in two ways. One approach is localization/detection of spikes, which provides not only the prediction for the whole image but also additional information regarding the spatial location of the spikes. Another technique is semantic segmentation (pixel-wise segmentation), which understands an image at pixel level. It enables dense predictions inferring labels of every pixel in the image, so that each pixel is labeled as an ear or background. Inspired by the success of the recent deep learning algorithms in computer vision applications, we propose a CNN approach combined with a superpixels technique known as simple linear iterative clustering (SLIC) (Achanta et al., 2010). The core idea is to overcome the computational complexity by using SLIC to generate homogeneous regions instead of processing at a pixel level. The homogeneous regions generated by SLIC will contain more information about the color and texture and are less sensitive to noise as opposed to pixel-level analysis. It also reduces the complexity of subsequent ear detection and localization tasks. The generated regions are later used as input data for the CNNs. The network is capable of not only recognizing spikes but also delineating the boundaries of each spike with the canopy based on dense pixel level predictions. **Figure 1** illustrates an end-to-end wheat ear quantification, including the offline training and online ear segmentation and counting. In the following section, we will describe the data collection/annotation process and the model architecture developed to localize wheat spikes within images and quantify them.

### Experimental Materials

The experiments were carried out at Rothamsted Research, UK (51°48′34.56′′N, 0°21′22.68′′W) in two fields, Great Field (Field Scanalyzer area) and Black Horse. Two experiments were conducted under the Field Scanalyzer platform (Virlet et al., 2016) during the growing season in 2014–2015 (hereafter referred to as 2015-FS dataset) and 2015–2016 (hereafter referred to as 2016-FS dataset). Six wheat cultivars (*Triticum aestivum* L. cv. Avalon, Cadenza, Crusoe, Gatsby, Soissons, and Maris Widgeon) were sown on 6th November 2014 and 20th October 2015 at a planting density of 350 seeds/m^2^. Nitrogen (N) treatments were applied as ammonium nitrate in the spring at rates of 0 kgN.ha^−1^ (residual soil N; N1), 100 kgN.ha^−1^ (N2), and 200 kgN.ha^−1^ (N3) for both years and 350 kgN.ha^−1^ (N4, 2015-FS only). The plot sizes were 3 × 1 m in 2015-FS and 2 × 1 m in 2016-FS.

The third experiment has been funded by DEFRA since 2008, known as WGIN (Wheat Genetic Improvement Network), to provide genetic and molecular resources for research in other DEFRA projects and for a wide range of wheat research projects in the United Kingdom. In this study, we collected images from the 2015–2016 experiment (hereafter referred to as 2016-WGIN dataset) at Black Horse field. Thirty wheat cultivars were grown at four nitrogen fertilizer treatments (N1, N2, N3, and N4), sown on 12th October 2015. Each repetition consists of a 9 × 3 m “main plot” and a 2.5 × 3 m “sampling plot” used for nondestructive measurement and destructive sampling, respectively. The three experiments in this study use a split plot design (with three blocks) and were managed by local agronomic practices.

### Image Acquisition

The images were acquired under conditions of natural illumination at multiple stages of ear maturation with different canopy complexities achieved through varied nitrogen inputs. The tests were carried out in extreme lightning conditions with typical environmental challenges faced in the field for images taken by different cameras and optics with no direct scaling relationships. **Table 1** summarizes the characteristics of the three trials carried out in this study. The camera models include different types of commercially available visible cameras with various spatial resolutions and configurations (**Table 1**).

**Table 1:** Characteristics of the three experiments considered in this study.

Dataset	Plot	Nitrogen (kg/ha)	Image	Camera	Image size	Focal length	Resolution (mm)	Date
**2015-FS**	72	0, 100, 200, 350	72	Prosilica GT 3300 Allied Vision	3,296 × 2,474	50 mm	0.22–0.29	13/07/2015
**2016-FS**	54	0, 100, 200	54	Prosilica GT 3300 Allied Vision	3,296 × 2,474	50 mm	0.26	29/06/2016
**2016-WGIN**	360	0, 100, 200, 350	78	Canon G12	3,648 × 2,736	6 mm	0.21–0.31	13/06/2016
			121	SONY-NEX-7	6,000 × 3,376	18 mm	0.14–0.25	13/06/2016

The images for 2015-FS and 2016-FS were collected by the Scanalyzer onboard visible camera (color 12-bit Prosilica GT3300) at a resolution of 3,296 × 2,472 pixels. The camera is positioned perpendicular to the ground and was set up at a fixed distance to the ground (3.5 m) for the 2015-FS experiment and at a fixed distance to the top of the canopy (2.5 m) for the 2016-FS. The camera is set up in auto-exposure mode to compensate for outdoor lighting changes.

**Figure 2:**
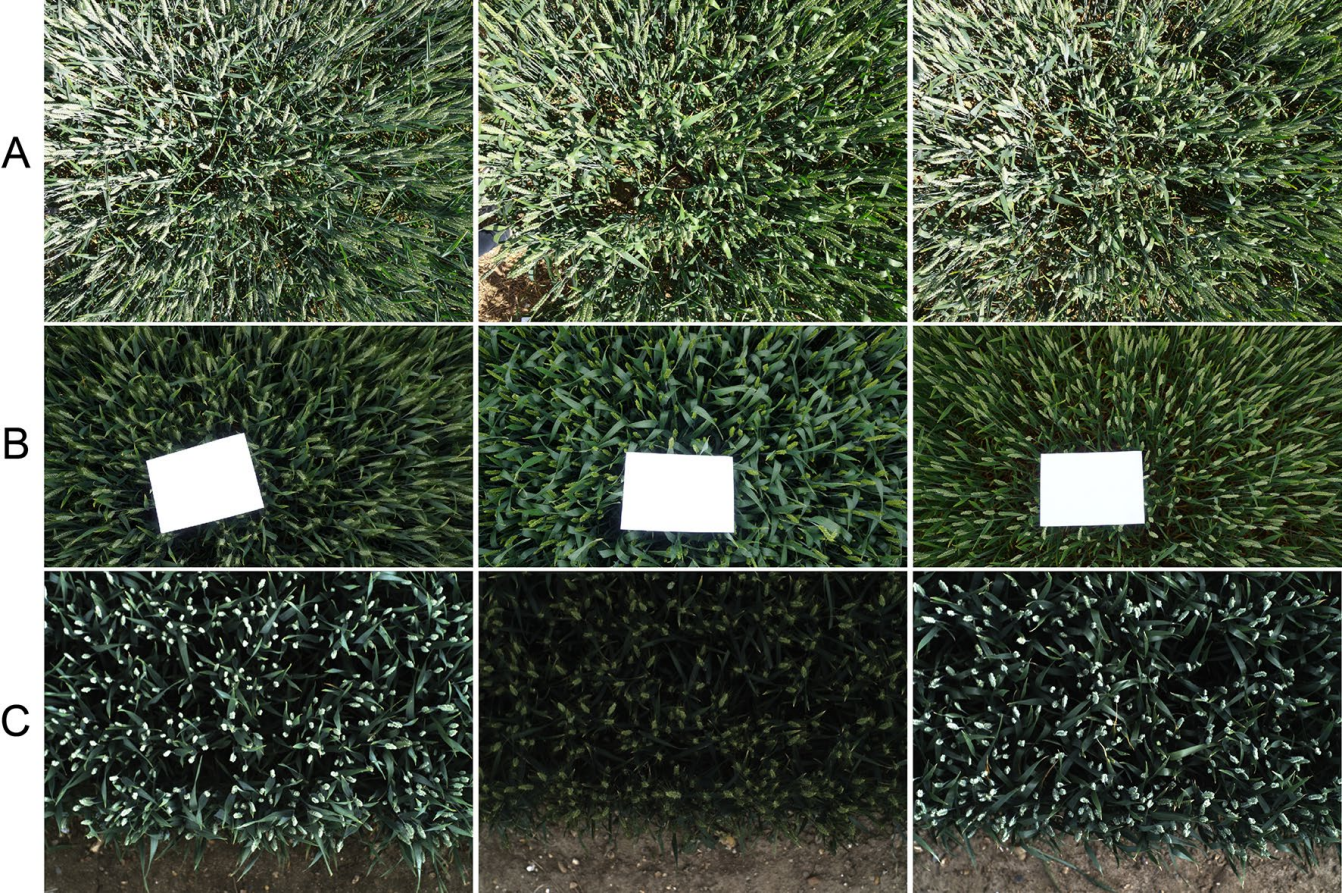
Overhead view digital images of wheat cultivars with different canopy complexities taken in the field using the handheld DSLR camera **(A** and **B)** and the Field Scanalyzer platform **(C)**. An A4 sheet is placed over the canopy for each image as a ground standard system to transform the total number of wheat ears in the image into the number of ears per square meter.

In the 2016-WGIN experiment, two handheld cameras, Canon G12 and Sony Nex-7, were used to acquire visible images with the resolution of 3,648 × 2,736, and 6,000 × 3,376 pixels, respectively (**Table 1**). Similarly, to the Field Scanalyzer, the cameras were set up in an auto-exposure mode and held vertically over the canopy. In addition, a rapid and easy ground standard system was implemented by placing an A4 sheet over the canopy in the field of view of the camera lens (**Figure 2B**). The ground system was used to transform the total number of wheat ears within an image into the number of ears per square meter.

### Evaluation

Two different evaluation methods were used and compared with the automatic ear-counting techniques. The first method is based on manual image-based annotation in which ears are manually counted on the images acquired by the Field Scanalyzer platform (2015-FS and 2016-FS datasets). Wheat ears were interactively marked using the VIA image annotator (Dutta et al., 2016), which enabled the automatic printing of the incremental number on each individual ear.

The second ground-truthing method is based on field manual measurements carried out for all three experiments. In the 2015-FS and 2016-FS experiments, ears were manually counted on six rows of 1-m length, corresponding to the 1-m^2^ area, for each plot. In the 2016-WGIN trial, the number of ears per square meter was estimated based on the method presented in Pask et al. (2012). Samples of four rows of 1-meter length were cut at anthesis, then the ears per square meter were derived from the aboveground biomass (ABG) and the dry weight (DW) of the fertile culm:

$${\text{Ears/m}}^{\text{2}}=\text{AGB }(\text{g}/{\text{m}}^{2})/\text{DW}\_\text{fertile culm (g)}$$

**Figure 2** shows the representation of digital images of different wheat traits taken under the Field Scanalyzer platform (**Figure 2C**) and a handheld DSLR camera (**Figures 2A, B**). As depicted in the sample images, the data were collected in different weather conditions, with illumination changes from cultivars with differences in ear shapes and sizes.

### Annotation and Generating the Training Dataset

The fundamental part of any supervised decision-making system, such as CNN, is how to specify the output based on a given set of inputs or training dataset. In practice, hundreds or even thousands of annotated training datasets are required to make a good training of CNN. Even though high-throughput image-based plant phenotyping systems like Field Scanalyzer (Virlet et al., 2016) exist and generate a huge amount of image data daily, a large set of annotated images with ground-truth are not widely accessible yet within the plant phenotyping community.

**Figure 3:**
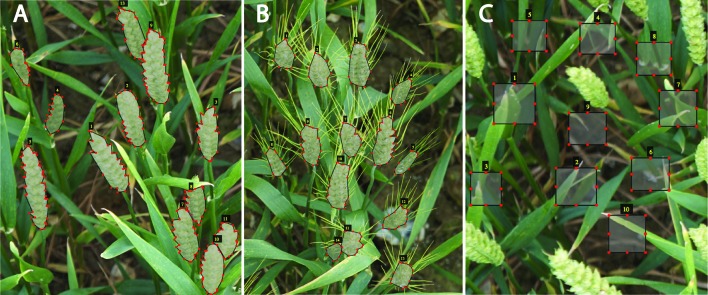
Training patches. Examples of expert annotation of spikes for different wheat cultivars without awns **(A)**, with awns **(B)**, and backgrounds (e.g., soil, leaves) **(C)**.

To expose our CNN model to a wider variety of images, the data were collected by a handheld DSLR Canon Camera with a resolution of 5,760 × 3,840 pixels from diverse Limagrain field trials at different stages from heading to maturation under different ambient illumination condition. The broad range of images enabled the constitution of a “strong” training dataset, covering the ears development from multiple wheat varieties, making the detection model more robust and thereby increasing the precision of the wheat spikes quantification. The graphical image annotation tool, VGG image annotator (VIA) (Dutta et al., 2016), was used to draw boxes around the background, such as leaves, stems, and soil, (**Figure 3C**), and draw strokes using the polygon tool around ears (**Figures 3A, B**). Here, 330 representative wheat images are selected to build the annotated training dataset, in which the illumination variations, weather conditions, wheat ears shapes, and reproductive stages are all considered. As a result, 24,938 ears and 30,639 backgrounds are manually annotated.

**Figure 4:**
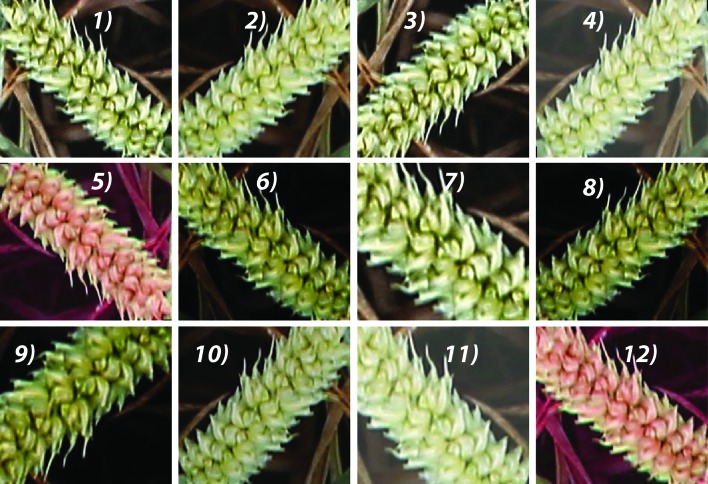
Augmented samples of the same spike with various transformations, such as random zoom, rotation, flipping, brightness, and gamma correction. For example, 1) the original image; 5 and 12) adjusted HSV color image; 6, 8, and 9) gamma color correction. 2-4, 7, 10, and 11) adjusted brightness samples Cropping, flipping, zooming, and scaling were applied to all images randomly with the probability of 0.5.

The next step is to combat the high expense of creating a training source with their corresponding labels. The augmentation model is constructed to simulate the illumination change by adjusting the HSV color space and applying various transformations, such as random rotation, cropping, flipping, zooming, scaling, and brightness to the images that are already in the training dataset (**Figure 4**). In addition, a nonlinear operation known as gamma correction (also referred to as gamma encoding or gamma compression) (Rahman et al., 2016) was applied to encode and decode luminance in the images. The augmented images are appended to the existing training samples, from which 20% of the sample set is randomly selected as the validation set (145,000 patches), and the remaining 80% is selected as the training set (580,000 patches; 300,000 ears and 280,000 backgrounds).

### Superpixels Segmentation

Most computer vision algorithms use pixel grid as the underlying representation of an image. However, grids of pixels do not hold a semantic meaning of an image nor represent a natural representation of a visual scene. It would be more efficient to work with perceptually meaningful entities obtained from a low-level grouping process. Superpixel algorithms aim to group pixels into perceptually meaningful regions based on their similarity characteristics, such as color and texture distributions. Superpixel techniques will reduce the complexity of images from thousands to millions of pixels to only a few hundred superpixels; thereby, it will diminish the influence of noise and potentially improves the computational efficiency of vision algorithms.

In light of the fundamental importance of superpixel algorithms in computer vision, many algorithms have been proposed in the literature (Achanta et al., 2010; Achanta et al., 2012, Li and Chen, 2015; Tu et al., 2018). The superpixel segmentation algorithms can be broadly categorized as graph-based segmentation and clustering-based segmentation. In graph-based techniques, an image is considered a planar graph, where pixel vertices and pixel affinities are computed for connected pixels (Felzenszwalb and Huttenlocher, 2004; Ren and Malik, 2005). Alternatively, the clustering-based method starts with a rough initial clustering of pixels, then the clusters are refined iteratively until some convergence criterion is met to form superpixels (Achanta et al., 2010; den Bergh et al., 2015; Achanta and Susstrunk, 2017).

**Figure 5:**
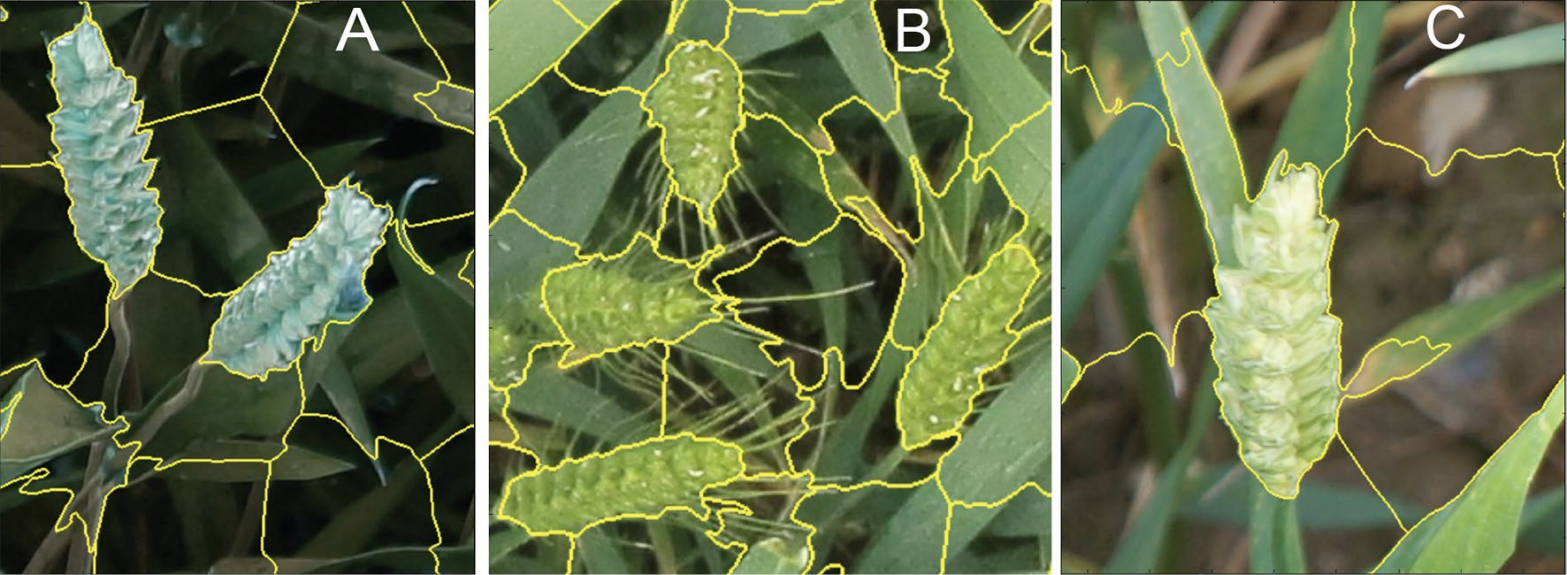
**(A**-**C)** Examples of superpixel segmentation using the SLIC technique. **C)** illustrates the imperfection in the SLIC method.

**Figure 6:**
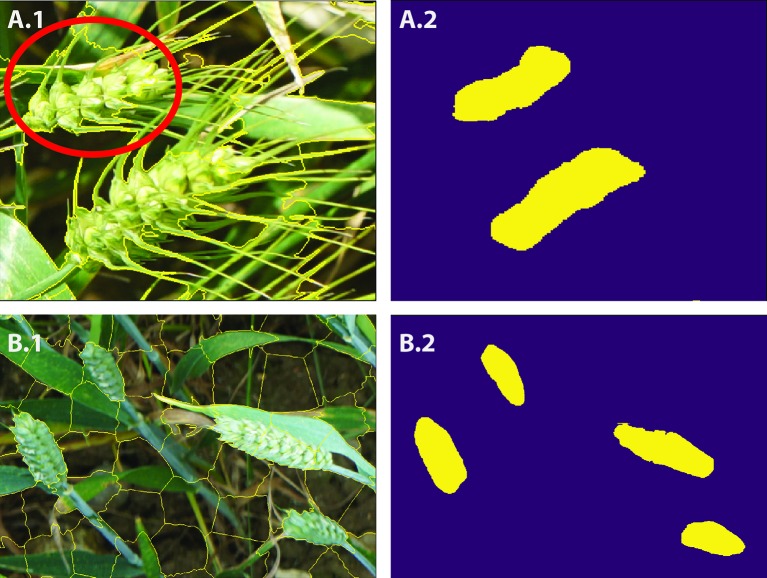
**A.1** and **B.1** show the SLIC superpixel outputs. **A.2** and **B.2** are the results of pixel-wise semantic segmentations. The red circle illustrates the imperfection in the SLIC method.

In this study, we use SLIC (Achanta et al., 2010; Achanta et al., 2012), which is fast and memory efficient for generating superpixels (Achanta et al., 2012). As opposed to other superpixels algorithms with many difficult-to-tune parameters, SLIC is simple to use in which the number of desired superpixels is its sole parameter. The spectral-spatial distance is measured between each pixel to its cluster center and then the cluster centers are updated using *K*-means clustering technique. For *N* prespecified superpixels, clustering pixels are represented based on their color similarity (CIELAB colour space) and pixel proximity in the 5-D space *C* *_i_* = [*l* *_i_* *, a* *_i_* *, b* *_i_* *, x* *_i_* *, y* *_i_*] where *i* = [1, *N*]. In this study, based on our experience, the number of superpixels is set to *N* = 3,000 to avoid oversegmentation and to produce roughly equally sized superpixels. We can also control the trade-off between the compactness of the superpixels and boundary adherence (Achanta et al., 2012). It means SLIC can prevent small or disconnected areas or islands within a larger region (**Figure 5** and **6A.1, B.1**). The candidate regions are then used as inputs for the CNN model to perform pixel-wise segmentation. Feeding the network with image descriptors extracted from the candidate regions enables the model to learn local information, such as texture and shape, rather than using the pixel grids.

### Architecture of the Convolutional Neural Network Model

As previously mentioned, SLIC reduces the computational complexity by partitioning an image into homogeneous regions, instead of extracting features at the pixel level (**Figure 5**). However, the SLIC method, like many other superpixel techniques (Felzenszwalb and Huttenlocher, 2004; Ren and Malik, 2005; Li and Chen, 2015; Wang et al., 2017), relies on handcrafted features, thus often fails to separate objects within an image in appropriate regions (**Figures 5C** and **6A.1**). To address the limitation, the proposed CNN model classifies each superpixel at a pixel level as opposed to characterizing the content of the entire candidate region and predict a single label. The network takes each candidate region as input data and outputs a pixel level segmented of the region (**Figures 6A.2, B.2**).

In general, semantic segmentation architecture in CNN can be broadly categorized as an encoder network followed by a decoder network. The encoder network gradually reduces the spatial dimension of the input by down-sampling and developing lower-resolution feature mappings, which are learned to be highly efficient at discriminating between classes. To get the dense pixel-wise classification, the decoder network semantically projects the discriminative features learned by the encoder onto the pixel space by up-sampling the feature representations into a full-resolution segmentation map. There are usually shortcut connections from encoder to decoder to help the decoder recover the object details better.

In this work, we leverage an existing model known as U-Net, which was originally designed for biomedical image segmentation for identifying lung nodules in a computed tomography (CT) scan (Ronneberger et al., 2015). The U-Net architecture consists of a contracting path to capture context and an asymmetric expanding path that enables precise localization. The model concatenates the encoder feature maps to up-sampled feature maps from the decoder at every stage. The concatenation allows the decoder at each stage to learn back relevant features that are lost when pooled in the encoder. Normally, U-Net is trained from scratch starting with randomly initialized weights (optimization variables). Since up-sampling in the decoder is a sparse operation, we need a good prior from earlier stages to better represent the localization.

Since transfer learning proved to be a powerful technique for semantic segmentation models, such as U-Net-like architectures (Iglovikov and Shvets, 2018), we used a pretrained VGG model (Simonyan and Zisserman, 2014) without fully connected layers as its encoder mechanism followed a decoder network as the original U-Net to further improve the performance of pixel level dense classification. The VGG family of CNN can be characterized by two components: 1) all convolutional layers in the network use 3 × 3 filters; and 2) multiple convolutional layer sets are stacking together before applying a pooling operation. Normally, the number of consecutive convolutional layers increases the deeper the network goes (Simonyan and Zisserman, 2014). The VGG-16 used in this work was proposed by a group of researchers in Oxford and the winner of the ImageNet competition (Deng et al., 2009) in 2013. It uses a stack of convolution layers with small receptive fields in the first layers instead of few layers with big receptive fields.

By using an existing architecture in which the weights are initialized on big datasets, such as ImageNet, the network can converge faster and learn more general filters. To construct the encoder, the fully connected layers were removed and replaced with a single convolutional layer of 512 channels that serves as a bottleneck part of the network to separate the encoder from the decoder. The network contains a total of four max-pooling layers. For each of the pooling layers, the spatial size of the feature map is reduced by a factor of 2 vertically and horizontally.

**Figure 7:**
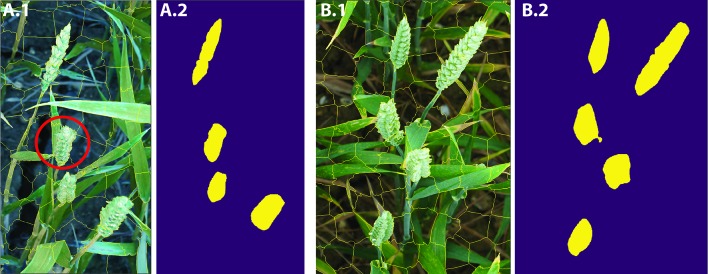
Encoder-decoder neural network architecture is also known as U-Net where VGG-16 neural network without fully connected layers as its encoder. The number of channels increases stage by stage on the left part while it decreases stage by stage on the right decoding part. The arrows show a transfer of information from each encoding layer and concatenating it to a corresponding decoding part.

**Figure 8:**
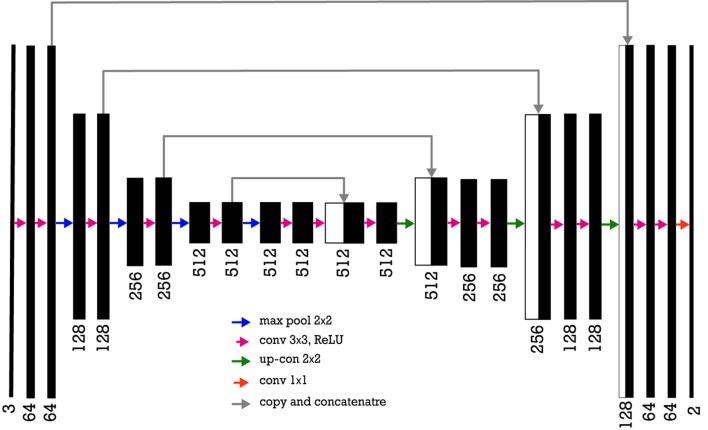
**A.1** and **B.1** show the SLIC superpixel outputs. **A.2** and **B.2** are the output of the *DeepCount* model. The red circle illustrates the imperfection in the SLIC method.

The decoder part of the network consists of up-sample and concatenation with an output of the corresponding part of the decoder followed by regular convolution operations (**Figure 7**). Since the pretrained VGG model takes an input of 224 × 224 pixels with three channels, the irregular superpixels need to be resized to achieve proper input into the model. The network takes superpixels as inputs and outputs a segmented version of the inputs. Each pixel is labeled as 1 (wheat spikes) or 0 (background), which generated a binary image (**Figure 8**). After the semantic segmentation, the median filter is applied to minimize the noise and remove the result of misclassification over the binary image. In this process, a window size of seven pixels slides over the entire image, pixel by pixel. Then, the pixel values from the window are sorted numerically and replaced with a median value of neighboring pixels. In the end, for contour quantification, a classical image processing algorithm known as the watershed technique is used for postprocessing for further segmentation of individual contour.

#### Loss Function

The role of the loss function in our parameterized learning was investigated. Parameterized learning will allow us to take sets of input data (ears and background) and their class labels and learn a function that maps the input to the output predictions by defining a set of parameters and optimizing over them. At a basic level, a loss function quantifies how good or bad a given predictor is at classifying the input data in our dataset (Marsland, 2009; Harrington, 2012).

**Figure 9:**
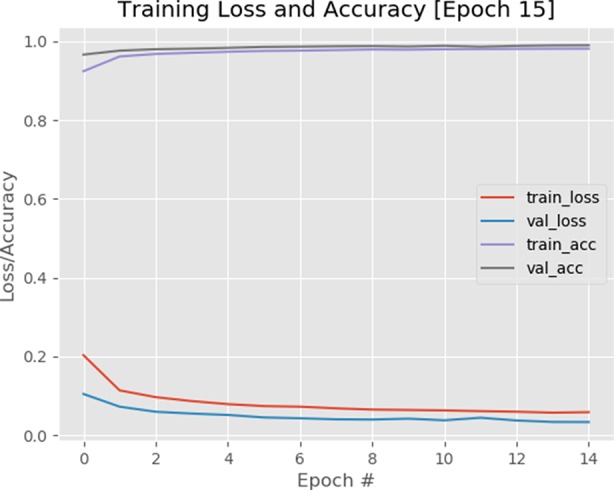
A plot of loss and accuracy throughout 15 epochs with a 1e-4 learning rate. Using of pretrained VGG model on ImageNet dataset helped the model to converge quicker.

The binary cross-entropy loss function is used to quantify how accurate the CNN method is at classifying the input data in our dataset (a brief overview of the cross-entropy loss function and the calculations are provided in the supplementary data). A visualization of the loss function plotted over time for our model is shown in **Figure 9A** visualization of training accuracy, training loss, validation accuracy, and validation loss plotted over time for the model is plotted after 15 epochs.^1^ The smaller the loss, the better a job the model/classifier is at modeling the relationship between the input data and output class labels. As shown in **Figure 9**, loss starts slightly high but then decreases rapidly and continues to stay low when trained on our dataset. As expected, the usage of the pretrained VGG model helps the network to converge faster, as a result, we obtained 98% accuracy after only 15 epochs. Furthermore, the training and validation curves match each other very closely, indicating that there is no issue of overfitting with the training process.

### Handcrafted Features Extraction Techniques for Wheat Ear Quantification

**Figure 10:**
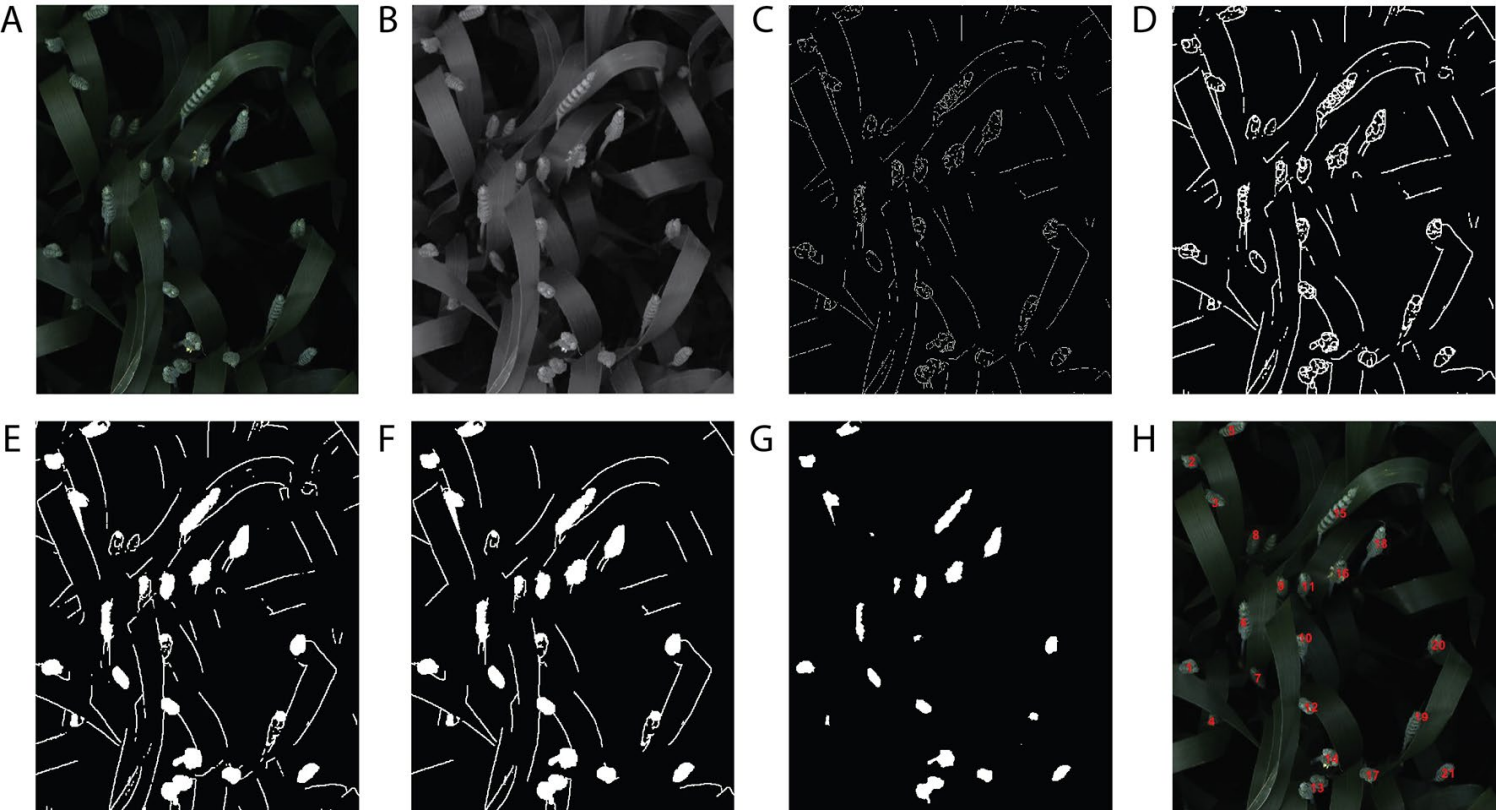
The handcrafted ear-counting method. **(A)** Original image. **(B)** Grayscale image. **(C)** Result after applying edge detection technique. **(D)** Dilate the image. **(E)** Fill the holes. **(F)** Filtering by removing small objects (noises). **(G)** Erode and smooth the image. **(H)** Counting the contours/ears.

A handcrafted image-based method presented in Jansen et al. (2015) was compared with the proposed *DeepCount* model. The technique is based on an edge detection technique and several morphological image-processing operations. First, the image is converted from a 3-D RGB image (**Figure 10A**) into a 2-D grayscale representation of the image (**Figure 10B**), then the edge detection based on Sobel kernel (Kaufman et al., 1994) performs a 2-D spatial gradient measurement on the gray image to emphasize regions of high spatial frequency that correspond to edges that return a binary image (**Figure 10C**). Edges may correspond to boundaries of an object, boundaries of shadowing or lighting conditions, and/or boundaries of parts within an object in an image. The next steps are morphological operations, including dilation to increase the size of foreground pixels (**Figure 10D**), which is useful for joining broken parts of the image. Filling the holes (**Figure 10E**) and removing small objects (**Figure 10F**) are the fifth and sixth steps. The final step is erosion, where pixels near the boundary of an object in the image will be discarded. A foreground pixel in the input image will be kept only if all pixels inside the structuring element are bigger than zero; otherwise, the pixels are set to zero (**Figure 10G**). In the end, a list of all contours is returned, and their numbers are printed out on the RGB image (**Figure 10H**). The handcrafted method will be referred to hereafter as the edge method.

## Results and Discussions

**Figure 11:**
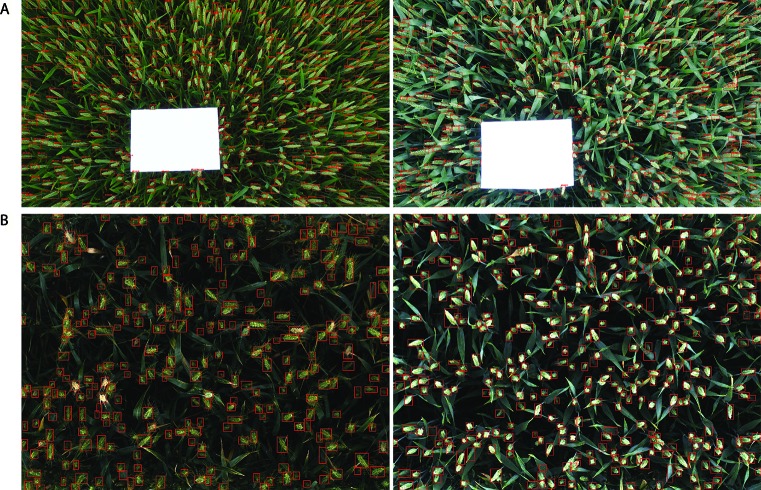
Examples of result images. **(A)** WGIN experiment with an A4 sheet used as a ground standard. **(B)** Field Scanalyzer experiment in 2015.

The performance of the proposed *DeepCount* model (**Figure 11**) was evaluated against the hand-engineered edge detection method and two manual evaluation techniques. The first technique was based on manual counting of ears within visible images while the second evaluation method was the field-based measurements. In addition, the ear-counting performances were quantified based on the coefficient of determination (R^2^), the root means squared error (RMSE), the relative RMSE (rRMSE), and the bias:

(1) $$\text{RMSE }=\sqrt{\frac{1}{N}{\sum \left(r_{i}-e_{i}\right)}^{2}}$$

(2) $$\text{rRMSE }=\sqrt{\frac{1}{N}\sum {\left(\frac{r_{i}-e_{i}}{r_{i}}\right)}^{2}}$$

(3) $$\text{Bias }=\frac{1}{N}\sum \left(r_{i}-e_{i}\right)$$

where *N* denotes the number of images, and *r* *_i_* and *e* *_i_* are the reference and estimated counts for image *i*, respectively.

The algorithm was tested on a workstation PC running a Centos7 operating system with 10-core Intel Xeon CPU, 3.6 GHz per CPU, 64 GB of memory, and Nvidia Quadro M5000 video card. The CNN framework was developed in python using OpenCV library and the Keras framework. While there is no restriction in the spatial resolution of the test images, the segmentation and quantification of wheat spikes will take approximately 90–100 seconds on a single image with the resolution of 6,000 × 3,376 pixels. The CUDA parallel acceleration was also used to improve the processing efficiency, especially for training the model. CUDA is a parallel computing platform created by NVIDIA, and the cuDNN library was developed for deep learning with GPU acceleration. The current method also has the potential to be faster in the future by CPU multithreading utilization.

### *DeepCount* Versus Handcrafted Edge Method

First, the performance of the automatic image-based methods (*DeepCount* and the handcrafted technique presented in section 2.7) was compared against manual image-based counting. In the image-based evaluation, 33,011 ears were manually counted from 126 images. The 2015-FS and 2016-FS trials include 72 and 54 images in which 22,284 and 10,727 ears were manually counted on the images, respectively.

**Figure 12:**
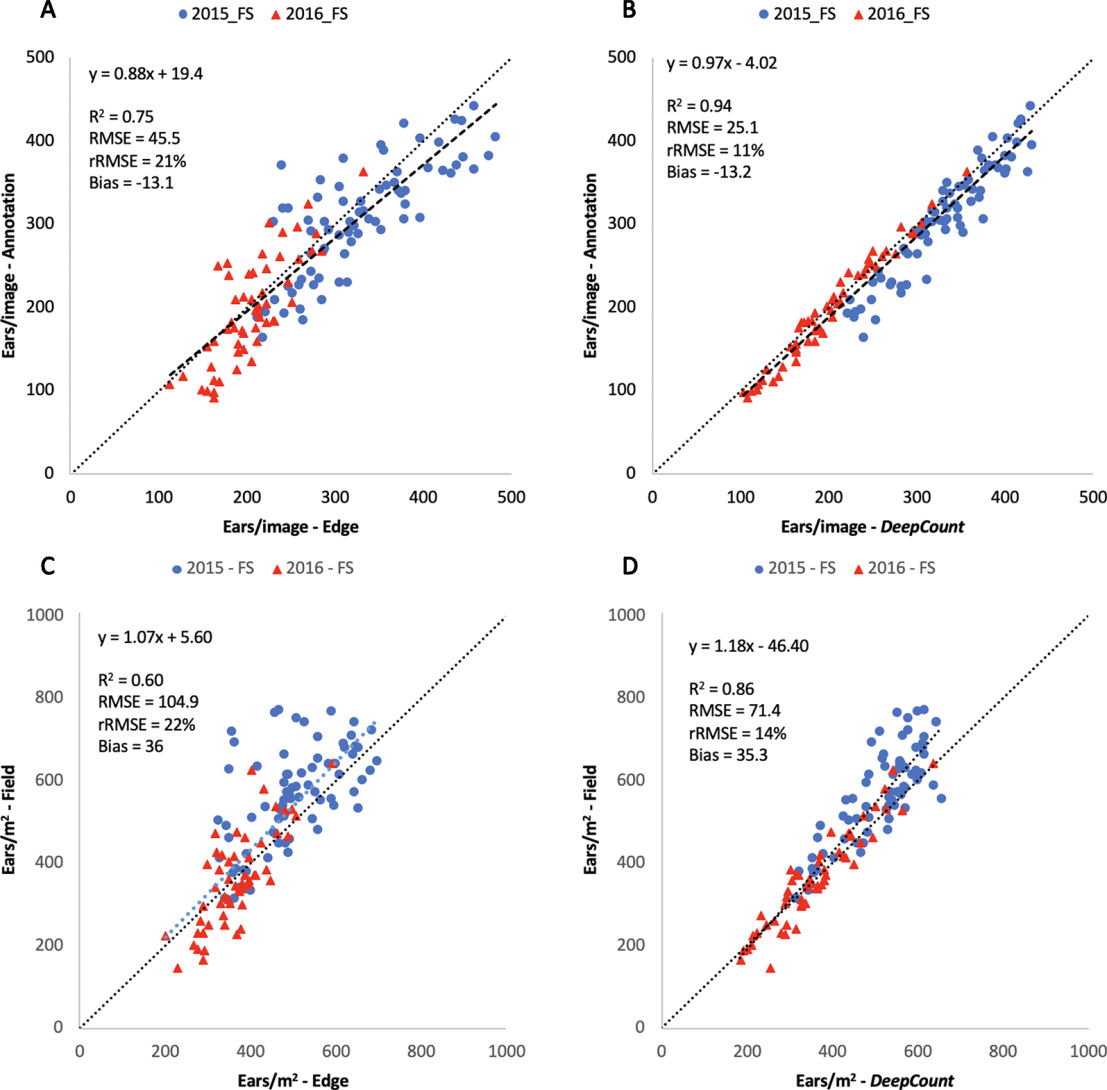
Comparison of the number of ears visually annotated on the images (Annotation – **A**, **B**) and the number of ears per meter square **(C**, **D)** with the number of ears estimated by the edge **(A**, **C)** and *DeepCount* **(B**, **D)** methods for the two datasets collected with the Field Scanalyzer in 2015 (blue dots) and 2016 (red triangles).

**Figures 12A, B** illustrate the linear regression between the automatic methods and the first evaluation method tested on the 126 images. The results showed a high correlation between the automatic methods and the manual image-based counting. The *DeepCount* model has a higher coefficient of determination and lower RMSE and rRMSE (R^2^ = 0.94, RMSE = 25.1, rRMSE = 11%) than the edge detection method (R^2^ = 0.75, RMSE = 45.5, rRMSE = 21%), indicating that the *DeepCount* technique was closer to the visual observation. In addition, the bias values of -13.1 and -13.2 for both methods show a slight overestimation of the number of ears compared to the visual assessment (**Figures 12A, B**).

The visual inspection of the results suggested that the edge method had more false positives than the *DeepCount* model. It was observed that in some cases, where leaves or objects have a clearer contrast than their surroundings, they were misidentified as ears. This was expected since the edge detection is defined as discontinuities in pixel intensity, in other words, a sharp difference and change in pixel values; thus, the edge detection method is more prone to noise. This may also pose more difficulties for the edge method to identify ears with awns (e.g., Soissons cv). The *DeepCount* model, on the other hand, had less false positive, regardless of the cultivars or level of nitrogen. Furthermore, visual inspection showed that the fraction of false negatives, in both automatic methods, appeared to be the failure of the watershed method to separate ears exposed to a severe degree of overlap.

While Fernandez-Gallego et al. (2018) argued that the edge method is unlikely to be reliable due to loss of RGB information during its color transformation to gray scale, our results indicated otherwise. The edge method showed similar performances compared to the method presented by the authors. The success rate metric (μ) used by the authors to evaluate the performance of their method showed 31.96–92.39% on RGB images and 65.36–93.01% on grayscale images, whereas we achieved a similar range of values with 86% and 81% in the 2015-FS and 2016-FS experiments, respectively. Moreover, the R^2^ values between the edge method and the two evaluation techniques (image-based counting and ground-based measurements) are high, with R^2^ = 0.75 and 0.60, respectively (**Figures 12A, C**). Nevertheless, the *DeepCount* model outperformed the edge method in every experiment carried out in this study. Our results are also in agreement with the method presented by Madec et al. (2019). The authors obtained R^2^ = 0.91 and rRMSE = 5.3% from their manual image-based ear counting, which is also very similar to the 2016-FS dataset, where the results showed R^2^ = 0.97 and rRMSE = 7% (**Figure S1**). We also found similar outcomes between our methods and the technique presented by Zhou et al. (2018); however, as the performance metrics differ, a quantitative comparison is not possible.

Furthermore, the performances of the edge and *DeepCount* methods were validated against the ground-based measurements after the numbers of ears were converted into ears per square meter. As shown in **Figures 12C, D**, the performance degraded slightly compared to the manual image-based measurements (**Figures 12A, B**). In the edge method, R^2^ reduced from 0.75 to 0.60, whereas the performance in the *DeepCount* model dropped from R^2^ = 0.94 to 0.86. The edge and *DeepCount* methods had a similar bias (36 and 35.3, respectively), which indicated that both methods underestimated the number of ears per square meter compared to the field data. In addition, the RMSE increased from 45.5 to 104.9 ears/m^2^ and 25.1 to 71.4 ears/m^2^ in both approaches, respectively.

A similar decrease in performance was also observed in Madec et al. (2019). This is partly attributed to the relatively different observation area used for the ground measurements and the visible images. The spatial representativeness was therefore limited to get an accurate comparison between the automatic counting and field-based measurements that were not measured at the same place over plots. For instance, in the 2015-FS trial, the ground-based measurements were obtained from six rows, including the edge rows; however, the same area was not taken by the Field Scanalyzer. The number of rows captured in the images varies between 3.5 and 5 rows (**Figure 2C**). An additional factor may also be due to the fact that some ears are hidden deep down inside canopies or partially visible on the borders of images, which pose more difficulties for the automatic models to identify them. Further improvement can be achieved between the automatic counting and direct counting in the field if the same protocol is followed by both methods during data acquisition. For example, in the 2016-FS trial, the results showed an improvement in performance when images were consistently taken from four middle rows in every plot (**Table 2**).

**Table 2:** Comparison between the number of ears per square meter counting from the field and the number of ears estimated by the *DeepCount* model for the three datasets collected separately and combined for each of the nitrogen levels. A and B are the slope and the offset of the regression line, respectively.

		N1	N2	N3	N4
2015-FS	A	1.16	0.96	0.64	0.68
	b	-18.40	55.22	263.06	282.56
	R^2^	0.58	0.46	0.15	0.22
	RMSE	61.50	60.30	92.20	122.90
	rRMSE	13%	10%	14%	17%
	Bias	42.30	35.20	58.90	100.10
2016-FS	a	0.75	1.10	0.93	
	b	45.23	-16.13	39.29	
	R^2^	0.59	0.75	0.89	
	RMSE	41.00	43.80	32.40	
	rRMSE	22%	10%	7%	
	Bias	-19.60	20.20	9.70	
2016-WGIN	a		0.95	0.71	0.88
	b		33.87	189.27	89.62
	R^2^		0.42	0.41	0.63
	RMSE		67.10	98.30	72.00
	rRMSE		15%	17%	14%
	Bias		15.60	57.90	30.80
All datasets	a	1.28	1.06	0.83	0.96
	b	-76.43	-4.10	131.26	66.39
	R^2^	0.81	0.69	0.53	0.60
	RMSE	52.20	61.40	90.00	84.40
	rRMSE	18%	13%	16%	15%
	Bias	11.30	20.70	50.30	44.30

### *DeepCount* Model Versus Field-Based Measurements

**Figure 13:**
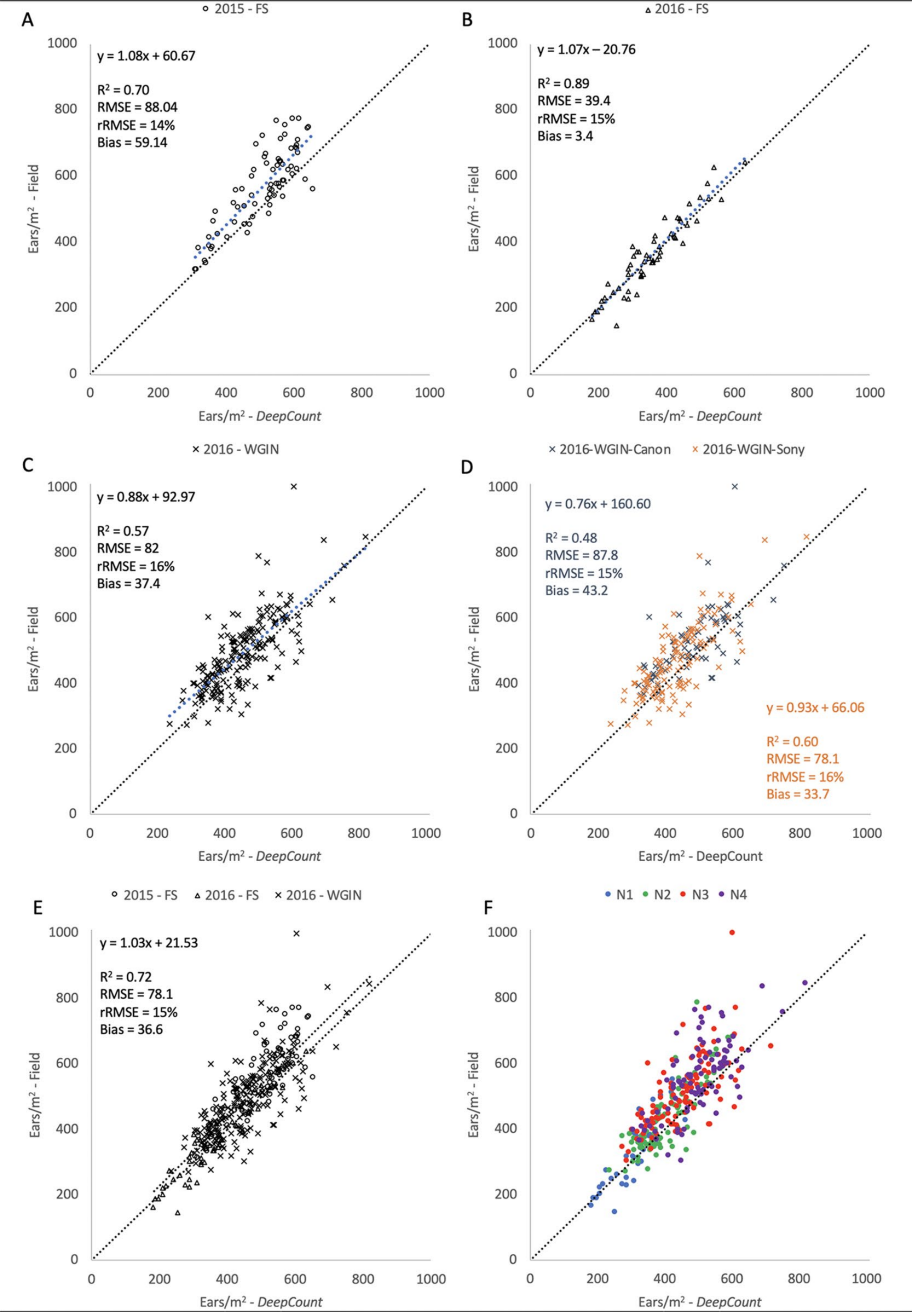
Comparison between the number of ears per square meter counting from the field and the number of ears estimated by the DeepCount model for the datasets collected with the Field Scanalyzer in 2015 (**A** – open circle) and in 2016 (**B** - open triangles), for the WGIN trial in 2016 (**C** – cross) separated by camera **(D)**, for all datasets together **(E)**, and for all datasets together separated by nitrogen level (**F** - N1: blue, N2: green, N3: red, and N4: purple).

The performance of the *DeepCount* model was further evaluated against the ground-based measurements in each individual trial and all together. As shown in **Figure 13**, the coefficient of determination was higher in the 2016-FS experiment (R^2^ = 0.89) compared to the 2015-FS (R^2^ = 0.70) and 2016-WGIN (R^2^ = 0.57) trials. Also, the lowest bias was obtained in the 2016-FS (bias = 3.6), followed by 2016-WGIN and 2015-FS with 37.4 and 59.14, respectively. As mentioned in the previous section, the notable difference in bias between the 2016-FS and the other trials may reside in the fact that, first, the measurements on the field and the visible images were obtained from the same area; also, in the 2016-FS, the camera was set up at a fixed distance to the top of a canopy (2.5 m) regardless of the height of the plots. As opposed to the 2015-FS trial, where the camera was set up at a fixed distance to the ground (3.5 m), or in the 2016-WGIN trial, where the distance between the handheld cameras and top of canopies vary from one plot to another.

Furthermore, the lower performance in the 2016-WGIN trial may be associated with several factors. First, improper placement of an A4 sheet used as a ground standard to transform the total number of wheat ears in an image into the number of ears per square meter. To have an accurate ear density estimation, the sheet should be placed perpendicular to the handheld camera’s viewing angle, which was not the case in many images taken from the WGIN-2016 trial. In addition, in some images, the ground standard was partially obstructed by leaves and wheat ears. Second, the perspective of the images may also account for the slight lack of correlation between the proposed model and the field measurements. While focal length does not change perspective per se, it does change how the ears are represented; thus, it is important to capture the scene optimally. The ultra-wide angle focal length used to capture images from 2016-WGIN (6 and 18 mm) provided a bigger field of coverage but caused a perspective distortion, particularly on the image borders. Last but not least, the manual field measurements may have introduced human error into obtained data.

Despite the above uncertainties, the *DeepCount* algorithm showed the same accuracy in every experiment (rRMSE = 15% ± 1) regardless of the number of ears identified in the images (2015-FS: 309–655, 2016-FS: 183–634, 2016-WGIN: 238–821) and types of cameras with different spatial resolutions. The same accuracy was also obtained when all three experiments were combined together (R^2^ = 0.72 and rRMSE = 15%). As shown in **Table 1**, two cameras (Canon and Sony) with different spatial resolutions and lens focal lengths were used to acquire images. In the Canon camera, we observed lower R^2^ but higher bias compared to the Sony camera (R2 = 0.48 and 0.60, respectively; bias = 43.2 and 33.7, respectively; **Figure 13C**); nevertheless, both show similar rRMSE (15% and 16%, respectively; **Figure 13C**). **Figure 13C** depicted outliers for both cameras, but it is not possible to attribute them to one of the cameras or a human error.

Overall, the *DeepCount* algorithm showed a solid performance in identifying wheat spikes at early or later growth stages. Visual inspection of results also showed that the proposed CNN model was able to discriminate ears and background (soil, leaves, etc.) and classified them on a pixel level. The proposed model was capable of minimizing effects related to brightness, shadow, ear size and shape, awn or awnless cultivars, and even overlap ears in most scenarios. It should be highlighted that the strength of the algorithm also resides in its training dataset, where images were collected by a third party on completely independent trials, different spatial resolutions, and different varieties than the wheat materials in this study. An improvement in the performance would be expected *via* the optimization of data acquisition process both in the field and within images. We believe that the optimum configuration is to take images at 2.0–2.5 m above canopies using the focal length between 35 and 60 mm, which is similar to what human eyes see. Moreover, we noticed that the textural information will fade away when spatial resolution is below 0.2–0.3 mm, which will degrade the identification performances.

### The Effect of Nitrogen Rate on the Performance of the *DeepCount* Model

We also investigated the effect of nitrogen on the performance of the *DeepCount* method. It was expected that the performance of the algorithm declines with the increase of nitrogen use since the canopies with a higher level of nitrogen have a higher ear density in which ears are more overlapped and clustered; however, the results showed otherwise. As depicted in **Table 2**, the overall N3 and N4 data had a lower R^2^ (0.53 and 0.60, respectively) compared to the overall N1 and N2 data (0.81 and 0.69, respectively). On the other hand, the 2016-FS and 2016-WGIN trials do not follow the same pattern. For instance, in the 2016-FS trial, N3 had the highest R^2^ value (R^2^ = 0.89), followed by N2 and N1 (R^2^ = 0.75 and 0.59, respectively), whereas in the 2016-WGIN, the N4 treatment had the highest R^2^ (0.63). Furthermore, on closer inspection, the N3 and N4 treatments showed the highest bias values and underestimation of the ear density in the 2015-FS, 2016-WGIN, and combined datasets.

Despite that, the accuracy of the overall experiments for each nitrogen treatment did not change too much as the rRMSE value for N1, N2, N3, and N4 were 18, 13, 16, and 15%, respectively. In the end, the results did not suggest that the performance of the *DeepCount* model degrades due to the complex canopies with a high level of ear density.

## Conclusion

In this study, the main objective was to present an automatic model that quantifies the number of wheat ears in an image or image series. Regardless of the challenges posed by the acquisition protocol or environmental variations in the field, the model was able to deliver the total number of wheat ears within an image and/or estimated the number of ears per square meter if a ground standard was present in the image. We demonstrated the feasibility of the proposed technique in which the model was validated on numerous images taken from a broad range of spatial resolution images and various data acquisition systems. It has been shown that the model can be an essential tool for high-throughput analysis and has the potential to reduce labor involvement considerably. To minimize the uncertainties between the automatic methods and the ground-based measurements, we recommend to 1) have the same sample areas, 2) have a more reliable ground standard rather than an A4 sheet used in this study, 3) take samples from a larger area for both image sampling and field measurements, 4) increase the spatial resolution of visible image to avoid losing the textural information, and 5) use the focal length of lens between 35 and 60 mm. The code can be found at https://github.com/pouriast. 

In the end, the aim is to increase the adoption of the approach by farmers and breeders by lowering the expense of camera equipment. The proposed model can be used as a high-throughput post processing method to quantify the number of spikes for large-scale breeding programs. Furthermore, the automatic technique can facilitate farmers to make improved yield estimates, which can be used to plan requirements for grain harvest, transport, and storage. Subsequently, improved estimates could reduce post farm gate costs.

The *DeepCount* model benefitted from the CNN architecture and even though the model was trained to distinguish two classes, nothing prevents modifying the network to classify and segment more plants or species. Given adequate training model, the proposed semantic segmentation technique offers the advantages of versatility and may be applied to other types of applications, such as segmenting different parts of plant organs and vegetation and even detect diseases. In future work, we aim to envisage the use of thermal and hyperspectral images, which will offer additional information to RGB visible images.

## Data Availability Statement

All datasets generated for this study are included in the manuscript, **Supplementary files** and DFW official website: https://ckan.grassroots.tools/

## Author Contributions

PS-T proposed and developed the computer vision methods. PS-T conducted the image processing analysis. NV performed the statistical analysis. NV planned and conducted the field experiments under the Scanalyzer. MH contributed to the revision of the manuscript and supervised the project. EA and PR provided the images to generate the training dataset used in this study. All authors gave final approval for publication.

## Funding

Rothamsted Research receives support from the Biotechnology and Biological Sciences Research Council (BBSRC) of the United Kingdom as part of the Designing Future Wheat (BB/P016855/1) and Defra Wheat Genetic Improvement Network (WGIN) (CH1090) projects.

## Conflict of Interest

The authors declare that the research was conducted in the absence of any commercial or financial relationships that could be construed as a potential conflict of interest.

## Abbreviations

FS, Field Scanalyzer; CNN, convolutional neural network; DNN, deep neural network; NN, neural network; SLIC, simple linear iterative clustering; WGIN, wheat genetic improvement network.

## Footnotes

1 Epoch is a hyperparameter which is defined before training a neural network learning model. It means the learning algorithm has seen each of the training data points *N* times.
