# Supplementary material for: DeepCount: In-Field Automatic Quantification of Wheat Spikes Using Simple Linear Iterative Clustering and Deep Convolutional Neural Networks
Source: Front Plant Sci. 2019 Sep 26;10:1176. doi: 10.3389/fpls.2019.01176 (PMC6775245; doi:10.3389/fpls.2019.01176)
Supplement: Supplementary file 1 [file DataSheet_1.docx]

**Gamma Correction:**

Gamma correction is also referred to as gamma encoding or gamma compression is a non-linear operation used to decode or encode luminance in digital images. In a digital camera there is a linear relationship between the number of photons hit the sensors of a camera and the signal receives; however, human perceive double the amount of light as only a fraction brighter which is a non-linear relationship. Moreover, human eyes are more sensitive to changes in dark tones that brighter tones.

In order to account a transition between the sensitivity of human eyes and sensors of a camera, gamma correction can be applied.

Gamma correction can be calculated as follows:

$$i_{output}=i_{input}^{(\frac{1}{G})}$$

Where G is the gamma value. If gamma values < 1, the image is shifted towards the darker end of the spectrum while gamma value > 1 will make the image appear lighter.
