# Supplementary figures and images for: DeepCount: In-Field Automatic Quantification of Wheat Spikes Using Simple Linear Iterative Clustering and Deep Convolutional Neural Networks
Source: Front Plant Sci. 2019 Sep 26;10:1176. doi: 10.3389/fpls.2019.01176 (PMC6775245; doi:10.3389/fpls.2019.01176)

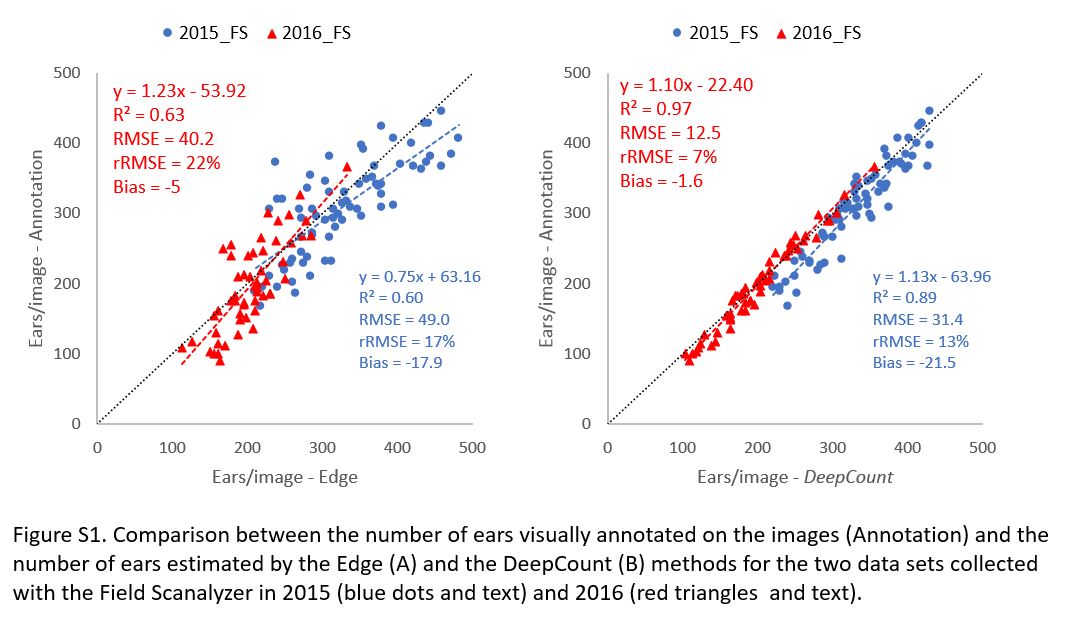

Supplement: Supplementary file 2 [file Image_1.jpeg]
