# Supplementary material for: DeepCount: In-Field Automatic Quantification of Wheat Spikes Using Simple Linear Iterative Clustering and Deep Convolutional Neural Networks
Source: Front Plant Sci. 2019 Sep 26;10:1176. doi: 10.3389/fpls.2019.01176 (PMC6775245; doi:10.3389/fpls.2019.01176)
Supplement: Supplementary file 3 [file Table_1.docx]

**Cross-entropy Loss and Softmax Classifiers**

Softmax classifiers are very popular in the context of deep learning and convolutional neural networks and are used in this study (section 3.6.1). Softmax classifiers give the probabilities for each class label, where it is much easier for humans to interpret probabilities, rather than margin scores as it is usually done in Multi-class SVM Loss. In cross-entropy loss, the mapping function *f* is defined such that it takes an input set of data *x_i_* and maps them to output class labels via dot product of the data *x_i_* and weight matric *W*:

$f\left( x_{i},W \right)=Wx_{i}$ (1)

The scoring function is defined as:

$s=f\left( x_{i},W \right)$ (2)

which implies that we can obtain the predicted score of the *j^th^* class via the *i^th^* data point:

$s_{j}=f\left( x_{i},W \right)_{j}$ (3)

The scores can then be interpreted as unnormalised log probabilities for each class label, which mounts to swapping out the hinge loss function with cross-entropy loss:

$L_{i}=-\log\left( e^{s_{y_{i}}}/\sum_{j} e^{s_{j}} \right)$ (4)

The loss function should minimise the negative log likelihood of the correct class:

$L_{i}=-\log P\left( Y=y_{i}|X=x_{i} \right)$ (5)

The probability statement can be interpreted as:

$P(Y=y_{i}|X=x_{i})=e^{s_{y_{i}}}/\sum_{j} e^{s_{j}}$ (6)

Where we use the standard scoring function form:

$s=f\left( x_{i},W \right)$ (7)

This yields the final loss function for a single data point, as above:

$L_{i}=-\log\left( e^{s_{y_{i}}}/\sum_{j} e^{s_{j}} \right)$ (8)

It should be noted that the algorithm here is actual base *e* (natural logarithm) since we are taking the inverse of the exponentiation over *e*. The actual exponentiation and normalisation via the sum of exponents is the *Softmax function*. The negative log yields the actual cross-entropy loss.

Finally, computing the cross-entropy loss over an entire dataset is done by taking the average:

$L=\frac{1}{N}\sum_{i=1}^{N} L_{i}$ (9)

We refer readers to(Marsland 2009; Harrington 2012; Rosebrock 2019) for more details about the concept of loss functions in machine learning.

Harrington P. Machine Learning in Action. 2012;5:384.

Marsland S. Machine Learning: An Algorithmic Perspective. 2009;

Rosebrock A. Deep Learning for Computer Vision. 2019;
